# Out of time, out of place: temporal desynchronization and gendered ageism in premature ovarian insufficiency

**DOI:** 10.1186/s12939-026-02954-4

**Published:** 2026-07-24

**Authors:** Johanna Budke

**Affiliations:** Interdisciplinary Center for Gender Studies (IZG), Bielefeld University, Universitätsstraße 25, 33615 Bielefeld, Germany

**Keywords:** Premature ovarian insufficiency, Gendered ageism, Age norms, Temporal dislocation, Feminist phenomenology, Qualitative research, Healthcare experiences

## Abstract

**Background:**

Premature ovarian insufficiency (POI) confronts cisgender women with menopause before the age of 40, placing them outside normative temporal expectations of the female life course. While its biomedical and psychosocial dimensions are well documented, less attention has been paid to how POI is shaped by age- and gender-based assumptions embedded in healthcare systems.

**Methods:**

This qualitative study employed a constructivist grounded theory approach (Charmaz), based on narrative interviews with eight women who entered menopause between the ages of 23 and 38, to examine how embodied experiences, social norms, and healthcare encounters —informed by feminist phenomenology—intersect in shaping interpretations of POI.

**Results:**

POI emerges as a profound temporal dislocation, with participants describing their bodies as ageing “out of sync” with their chronological age and social environment. This desynchronization disrupts biographical expectations and, in some accounts, crystallize into internalized ageism. Biomedical frameworks offer limited interpretive resources beyond deficit-oriented understandings, while healthcare encounters are frequently marked by misrecognition and epistemic injustice, as POI falls outside age-normative clinical expectations.

**Conclusion:**

The article suggests that POI can be read as a site of age- and gender-based misrecognition, in which ageism manifests less through overt discrimination than through internalization and structural invisibility. Addressing these inequalities requires more inclusive clinical frameworks, improved psychosocial support, and greater recognition of diverse temporalities of ageing across the life course.

## Background

Human life is often socially structured by a linear timeline that delineates clear phases such as youth, adulthood, and old age. Menopause is firmly anchored on this timeline as a biological and social marker of middle age [1]. For a significant number of women, however, this normative concept of time is fractured by premature ovarian insufficiency (POI)—a condition in which menopause occurs before the age of 40 [2]. POI affects approximately 3,5 − 3,7% of women under 40 [5], yet it remains significantly underrepresented in both clinical research and public discourse on menopause. POI has been shown to affect women across biological, psychological, and social domains, extending well beyond reproductive loss [7]. POI carries direct biological consequences of early estrogen deficiency, including accelerated bone loss, increased cardiovascular risk, and effects on sexual health such as vaginal dryness and reduced libido [8]. Studies also highlight the role of bodily symptoms (hot flushes, cognitive changes, fatigue, and altered physical appearance) in shaping self-perception and social participation [9, 3]. Research has documented experiences of social isolation, a disrupted sense of identity, and difficulties in communicating the condition to partners, family members, and employers [10, 11]. Studies consistently report elevated rates of anxiety, depression, and reduced quality of life among affected women [12, 8]. This emotional burden often extends into close relationships, as partners must jointly navigate decisions around fertility, family planning, and disclosure to others, adding a distinctly relational dimension to the experience of diagnosis [9, 10, 3, 13].

Diagnosis is often delayed by several years, partly because the condition falls outside dominant assumptions about who menopause affects and when [6] and partly for clinical reasons: intermittent ovarian activity can still occur in POI, occasionally resulting in spontaneous ovulation or pregnancy, and there remains ongoing debate over the precise diagnostic criteria, both of which complicate timely diagnosis [8]. The diagnosis is frequently experienced as a traumatic shock, particularly when it is unexpected and when the aetiology remains unclear—as is the case in the majority of idiopathic presentations [6, 14].

This distress is compounded by how the condition has predominantly been conceptualized in existing research. The loss of reproductive capacity is treated as the central source of distress, shaping both clinical attention and support provision [14–16]. While this focus is understandable given the significance of fertility for many affected women, it simultaneously narrows the frame of inquiry and risks reducing a complex lived experience to a single dimension.

Women with POI frequently report feeling that their experience is not recognized or taken seriously within healthcare encounters, contributing to a sense of being unseen within a system largely oriented toward women in normative reproductive trajectories and age-expected life stages [17, 15].

Underlying both this relational and institutional invisibility is a more fundamental disruption of time itself. This premature cessation of ovarian function disrupts the expected sequence of life events, creating a profound conflict between lived bodily experiences, chronological age, and social expectations [3, 4]. Notably, the perception of premature aging has recently been identified as one of the most salient themes across the qualitative literature on POI, second only to fertility loss [7]. Yet the ways in which these age-related dimensions of POI are structurally produced and normatively evaluated—including how they are shaped by gender—remain comparatively underexplored. This article addresses that gap by examining how women with POI experience and negotiate age-related expectations, both in their everyday lives and in their encounters with the healthcare system.

### Theoretical framework

This temporal dimension is theorized here through the sociological concept of age norms and age expectations: the socially shared expectations that certain life events should occur at particular times and in a particular sequence [18]. Age norms are not neutral descriptors of typical patterns; they function as normative prescriptions that define what counts as “on time” and “off time” in the life course, attaching social meanings and evaluative judgements to deviations from expected sequences. Menopause, in most cultural contexts, is firmly coded as an “on time” event for women in their late forties and fifties, a transition associated with specific social positions, relationships, and bodily expectations [19]. When menopause occurs decades earlier, it constitutes a profound “off-time” experience that exposes affected women to the normative gaze: they are positioned as too young for what is happening to their bodies, while these bodily processes are culturally coded as belonging to later life [3]. This desynchronisation between biological state and age-normative expectations generates a specific form of social vulnerability, one that is not simply about illness but about being out of place in the temporal order of gendered life.

To account for the bodily and subjective dimensions of this experience, this study draws on feminist phenomenology as a complementary theoretical resource. The phenomenological tradition, developed most influentially in relation to the lived body by Merleau-Ponty (1945) [20], begins from the insight that perception is not a purely cognitive act but a bodily one: we do not merely think the world, we inhabit it through our bodies. Merleau-Ponty distinguishes between the body as object—measurable, observable from the outside—and the lived body (Leib), the body as experienced from within, as the medium through which we orient ourselves in space, time, and social life. The lived body is, in his terms, the “vehicle of being in the world” (20:106): it is always already engaged with its surroundings, and this engagement is affective and habitual as much as it is conscious. Crucially, the lived body is also intersubjective: it is formed in relation to others and to the social world, carrying within it the traces of cultural norms and shared meanings, often below the threshold of deliberate reflection.

Feminist phenomenology, developed through the work of Beauvoir (1949) [21] and extended by Young (1980) [22] and others, builds on this framework by foregrounding the ways in which the lived body is always already a gendered body [23]. Young’s analysis shows how women in patriarchal societies experience their embodied selves as constrained, observed, and subject to normative judgement in ways that men typically do not. Feminist phenomenology further distinguishes itself by attending to marginalized and non-normative bodily experiences—those that fall outside the dominant cultural scripts of what a body should do, look like, or be capable of at a given point in life. As Folkmarson Käll and Zeiler ([24]:7) note, this approach “brings out the complexities of experiences that deviate and are excluded from the realms of normality.” Fisher [25] extends this further by emphasizing that the lived body does not only encounter others, but is embedded in a wider sociopolitical context characterized by asymmetrical power relations and normative frameworks that shape what kinds of experience are possible, legible, and supported. Applied to POI, this perspective makes it possible to examine how the hormonal and physical changes of early menopause are not experienced in a vacuum but are always already interpreted through culturally available frameworks—frameworks that tend to equate female bodily value with youth, fertility, and vitality. The body that is “too early” menopausal thus becomes a body experienced as failing, out of time, and difficult to inhabit within existing social scripts of gendered selfhood—while simultaneously finding itself structurally unsupported within healthcare institutions organized around different normative assumptions.

While the general psychological burden of POI is well-documented [7], and existing research has examined its implications for femininity and identity [16], this body of work has rarely brought age normativity and feminist phenomenology together as a combined interpretive lens.

In a society that often idealizes youth, the premature confrontation with a condition associated with aging and its negative stereotypes creates a unique form of vulnerability [26]. Taken together, the concepts of age normativity and feminist phenomenology provide a multi-layered framework for examining this process: they allow us to attend simultaneously to the normative temporal structures that render early menopause an “off-time” event, and the bodily and affective dimensions of inhabiting a body that does not fit the social scripts of one’s age.

Building on this theoretical framework, the following section outlines how this study was designed and conducted.

## Methods

To explore the embodied and social dimensions of premature menopause, this study adopted an interpretive approach oriented toward the lived experience of those affected. Constructivist Grounded Theory Methodology [27] was employed as the overarching framework, guiding both data collection and analysis. This approach was selected for its capacity to generate conceptual understanding from experiential data while remaining sensitive to the social conditions that shape individual experience.

The analysis presented here builds on the temporality category—one of four foci identified in the author’s prior doctoral grounded theory analysis of POI—encompassing three dimensions: temporality in healthcare encounters, temporality in relation to unfulfilled desires for children, and temporal normative expectations regarding life courses [9]. The present article elaborates on the third of these dimensions, drawing on the sociological concept of age norms and age expectations [18] to theorize its normative structure.

Data were generated through narrative interviews [28] with eight cisgender women who had entered menopause between the ages of 23 and 38. Narrative interviewing was chosen to allow participants to recount their experiences in their own terms and temporal order, minimizing the imposition of predefined categories. Participants were invited to speak openly about the onset of menopause, their bodily experience, and the social contexts in which they navigate life with POI. Inclusion criteria were a diagnosis of idiopathic POI, residence in Germany, and sufficient German language skills to participate in an interview, together with the willingness to provide informed consent. Participants were excluded if they were unable to participate in an interview due to language or cognitive barriers, or in the absence of a POI diagnosis. The sample was purposefully constructed in line with theoretical sampling principles, with recruitment continuing until sufficient conceptual density was achieved. For instance, after the first two interviews foregrounded unfulfilled desires for children as a central concern, a subsequent participant whose reproductive plans were long since resolved was purposefully recruited to examine whether the emerging themes extended beyond this context. Further theoretical sampling decisions were guided by this logic of contrast, incorporating participants who varied in time since diagnosis, age, and additional chronic health conditions. An overview of participant characteristics is provided in Table 1.

**Table 1:** Participants characteristics

Pseudonym	Age at interview	Age at diagnosis	Relationship status	Parenthood
Nora	33	30	married	One child
Elena	35	28	married	One child
Natalie	32	31	married	Two children
Jenny	35	35	married	No child
Doreen	40	38	married	No child
Amaia	25	23	single	No child
Sarah	32	31	married	No child
Marie	33	24	married	One child

The eight interviews were conducted between November 2021 and June 2022. Seven interviews were conducted face-to-face; one was conducted via Zoom due to ongoing contact restrictions related to the COVID-19 pandemic. Interviews lasted 77 min on average (range: 31 min to 3 h 26 min) and were audio-recorded using a digital recording device. Recordings were subsequently transcribed verbatim. Most transcripts were prepared by a professional transcription service, with a subset transcribed directly by the researcher; transcripts produced externally were verified by the researcher, who listened to the audio recordings while reading the transcripts. Prior to analysis, transcripts were anonymized by replacing identifying details such as place names and the names of clinicians or children. All interviews were conducted in German; quotations presented in this article have been translated into English by the author.

Data analysis was organized and structured using the computer-assisted software f4analyse, which allowed for the color-coding of text segments and individual words, facilitating coding, memo-writing, and the overall organization of the data. The software also enabled cross-interview displays, which facilitated comparison across individual interviews.

Analysis followed an iterative, multi-stage coding process. During initial coding, the data were examined as closely as possible to the material itself, using word-by-word and line-by-line coding to capture both the content of what was said and the discursive manner in which it was articulated. Focused coding was subsequently applied to identify the most significant and recurrent codes, subjecting these to deeper analysis in order to uncover underlying structures and meanings. It was during this phase that the concept of gendered ageism began to take shape, as codes relating to age-based misrecognition, initially identified separately across participants’ accounts, were progressively linked and refined through memo-writing. This process built on the age-normative category identified in the author’s prior doctoral analysis, elaborating in particular how participants’ sense of falling outside the normatively expected ‘right time’ for menopause left them feeling unrecognized within dominant discourses on menopause, and how bodily symptoms and changes came to be interpreted through an ageing lens that was frequently experienced as negatively connoted rather than neutral. Throughout this process, analytical ideas were noted as memos, which served as a key instrument for developing and refining interpretive insights.

As this analysis drew on the coded dataset generated during the author’s prior doctoral research rather than involving new data collection, the renewed focused coding proceeded through a systematic re-examination of the existing corpus of codes and memos across all eight interviews. Conceptual density for the age-normative category was achieved across the sample as a whole; the more specific pattern of gendered ageism, however, did not emerge uniformly across all participants’ accounts, but could be traced, often implicitly rather than as an explicit theme, in four of the eight interviews (Doreen, Amaia, Elena, and Nora), as reflected in the illustrative extracts presented in the Results.

Reflexivity was addressed throughout the research process through the writing of memos and postscripts, the documentation of the researcher’s own assumptions and pre-understandings, and participation in analysis groups with other researchers. Prior to the original data collection for the author’s doctoral research, the researcher’s existing knowledge and assumptions regarding early menopause were mapped and documented. This pre-existing knowledge was concentrated primarily on the medical care context—diagnostic delay, the diagnostic moment as a point of crisis, hormone replacement therapy, gaps in psychosocial support—while temporal and age-related dimensions were largely absent from this initial framework. This imbalance was treated as a methodological caution: throughout data collection and coding, the researcher remained attentive to the risk of imposing this pre-existing relevance structure onto participants’ accounts, staying open to unanticipated dimensions such as age and life-course normativity. The analysis was further supported by ongoing engagement with an interdisciplinary analysis group within the Research Training Group ‘Experiencing Gender: Constitution and Transformation of Being in the World’ at Bielefeld University, whose members held backgrounds in sociology and gender studies. As the author’s own disciplinary background lies in public health, this exchange was particularly valuable in complicating a primarily public health-oriented reading of the data, drawing attention to sociological and gender-theoretical dimensions of participants’ accounts that might otherwise have remained underexamined. This methodological approach enabled analysis of how being-in-the-world is intertwined with social knowledge: the study illuminates which ways of thinking, feeling, and bodily practice are drawn upon as forms of knowledge in the experience of POI and which, conversely, appear unavailable or unliveable for the subjects concerned.

The study was reviewed by the Ethics Committee of Bielefeld University (application number 2021 − 197) and conducted in accordance with the ethical guidelines of the German Psychological Society (DGPs) and the Professional Association of German Psychologists (BDP).

All participants provided written informed consent after being informed about the study’s aims, data protection measures, and their right to withdraw at any time. Anonymity and confidentiality were strictly ensured, and all data were securely stored on encrypted devices.

This article was prepared in accordance with the Standards for Reporting Qualitative Research (SRQR) guidelines [29].

## Results

The experience of POI profoundly disrupts socially normative notions of life trajectories, particularly those concerning age. While medical literature defines POI as menopause occurring before the age of 40, highlighting its physical and emotional implications [2, 30], the qualitative data reveal how this incongruence is concretely lived and negotiated by women with POI in their everyday lives. This section presents findings from narrative interviews, illustrating how women with POI navigate this disruption and construct their age identity amidst conflicting temporalities.

An overview of the resulting categories, subcategories, and illustrative data extracts is provided in Table 2.

**Table 2:** Overview categories and subcategories

Category	Subcategory	Illustrative data extract
Temporal desynchronization	Mismatch with normative menopause narratives	*“So what am I supposed to do? […] how am I supposed to reinterpret that for myself?” (Amaia*,* l. 790)*
	“Too early” experience	*“I noticed that it’s a little*,* um*,* gray […] I thought*,* why already?” (Doreen*,* l. 675)*
Age-body dissonance	General bodily-age mismatch	*“I feel like a hundred-year-old*,* physically” (Nora*,* l. 191)*
	Chronological vs. hormonal age	*“The number says I’m young*,* but my body is getting older” (Amaia*,* l. 807)*
Institutional misrecognition	Structural unpreparedness	*“Menopause. Now? What? I’ve never heard of that.” (Nora*,* quoting clinician*,* l. 364)*
	Epistemic injustice in clinical encounters	*“I noticed how many people have no understanding of why one might go to a fertility clinic*,* or that something like early menopause even exists” (Nora*,* l. 366)*

### Mismatch with normative menopause narratives

Interviewees frequently expressed a disconnect between their experience of POI and prevailing societal narratives of menopause, which are often linked to middle age and specific life transitions. These narratives, as described by Amaia, typically associate menopause with autonomy, children leaving home, and the opportunity for self-realization. However, for women with POI, this template fails to align with their lived reality:

For me, it’s not a new phase of life where people say, ‘Oh, then they split up with their partner and start a new relationship or something,’ or ‘the kids have left home’—people always say that, ‘the kids have left home, finally some free time for me.’ Well, no, I… In my case, the kids haven’t left home. So what am I supposed to do? So, that’s—how am I supposed to reinterpret that for myself? (Amaia, lines 785–790)

Amaia’s statement underscores the struggle to reconcile personal circumstances with these normative expectations, revealing a significant gap between social ideals and her own life with POI. This deviation necessitates an effort to adapt, which is often perceived as difficult, as the condition “*is not actually supposed to happen yet, so to speak, and my life doesn’t really fit with it at all*” (Amaia, lines 555–557). This feeling of incongruity is closely tied to stereotypical images of menopause that young women like 25-year-old Amaia cannot integrate into their self-perception.

### The ‘too early’ experience: age-body dissonance

The concept of age-body dissonance emerged as a central theme, directly linking POI to age and producing a pronounced dissonance between participants’ bodily experience and their chronological age. Participants articulated normative expectations regarding the physical and emotional state appropriate for their chronological age, which were often unmet due to POI. Marie, 33 at the time of the interview, expressed the pressure to feel *“strong and age-appropriate (laughs frustrated) and not constantly tired and exhausted”* (Marie, lines 185–186), highlighting a perceived failure to meet societal demands for vitality at her age.

This sense of premature aging was further exacerbated by observable physical changes. Doreen, 40 at the time of the interview, described experiencing signs typically associated with later life, such as withered skin and graying hair, which she connected to her early menopause:

Because, well, I also noticed that my skin has kind of (laughs slightly) become more withered, right? That’s kind of—I mean (.) that’s kind of annoying, because I actually thought I always looked so young. \[…] And with my hair. I noticed that it’s a little, um, gray, and actually I noticed that as early as (.) yeah, at 33 or so, right? I thought, why already? That was kind of a thing. (Doreen, lines 669–675)

Doreen’s narrative reflects regret, surprise, and annoyance, stemming from the feeling that these changes are occurring “too early,” thus emphasizing the primary significance of temporality in her experience.

A further dimension of this age-body dissonance emerged in the account of Nora, 33 at the time of the interview, who described the physical pain accompanying her early menopause in strikingly age-coded terms: “*My whole body has been hurting since, well, since the birth, my feet extremely, but also my joints, and I feel like a hundred-year-old, physically*” (Nora, lines 189–191). Nora herself acknowledged difficulty in disentangling the effects of POI from those of recent childbirth and the physical demands of new motherhood. To describe her bodily state, Nora reaches for the image of extreme old age, associating this with pain and physical limitation.

Across these accounts, the normative expectations attached to age operate not only at the level of appearance or life stage, but at the level of bodily feeling itself. Marie’s formulation is telling in this respect: to feel “strong and age-appropriate” (line 185) is framed as an obligation, a standard that the menopausal body at thirty-two fails to meet.

These accounts illustrate how age-normative expectations shape participants’ bodily self-perception, linking functionality, productivity, and youth as closely associated markers of legitimacy and value.

A key finding was the distinction drawn by interviewees between “chronological age” and “hormonal age.” Participants, such as Elena (line 72), described a simultaneous experience of being chronologically young but hormonally old. This hormonal reality, immediately felt on a physical level, often overshadowed their chronological age in terms of perceived significance. Amaia articulated this dissonance when comparing herself to her friends:

And (.) then (.) I sometimes do think, yeah, um, when I see my friends, I think, yeah, they’re so much younger than me and you can really see their youth in them, and they can truly feel that and embrace it for themselves because, even if they feel ugly sometimes, they’re young and they know it. And for me, it’s somehow in question whether I’m still young, because sure, the number says I’m young, but my body is getting older, and it’s aging faster on some level than others’. That’s why it’s a reality that’s just there. (Amaia, lines 807–813)

Amaia’s account reflects a perceived asynchrony with her peers, whose youth she experiences as something she can no longer fully access or feel within herself. The “promises” associated with her chronological life phase—such as youthfulness and vitality—are unfulfilled due to her hormonal state. This leads to a profound sense of loss, as Amaia expressed:

And I’d really like to know what it feels like to be young (laughs frustrated) and truly, completely young? \[…] And sometimes I have this feeling that it was kind of taken away from me, that I’m missing such a—such a time in my life, such a phase, even though I’m actually in it, that it was still cut out, and I feel like someone pressed fast-forward, and I ended up somewhere else (Amaia, lines 666–673).

This feeling of being “fast-forwarded” into a later life stage highlights a key dynamic of the POI experience: the coexistence of competing temporal registers, in which embodied changes are often more salient than chronological age. Amaia does not deny that “the number says I’m young” (line 810)—but this carries little weight against bodily experiences that are read as signs of aging. Its force lies in its immediacy: it is felt, rather than calculated. This temporal dislocation, grounded in the immediacy of bodily experience, also shapes how women with POI encounter social institutions, including a healthcare system that, as the following section shows, is itself organized around very different assumptions about whose body ages when.

### Institutional misrecognition: encountering the healthcare system

A recurring theme across the interviews was the encounter with a healthcare system structurally unprepared for the existence of early menopause in young women. These encounters were not experienced as isolated failures of individual practitioners, but as symptomatic of a broader institutional blind spot, one in which the combination of youth and menopause simply does not compute.

This structural dimension becomes visible in Nora’s account of an encounter with an anesthesiologist at a university clinic, during a routine pre-operative consultation at a fertility clinic. The doctor, unfamiliar with the context, opened with a dismissive remark about social freezing — “*that’s not really my thing, I have no understanding for that*” (lines 360–361)— before proceeding to explain the procedure. When Nora clarified that she was not there for social freezing but because of early menopause, the doctor’s response was telling: “*Menopause. Now? What? I’ve never heard of that. I’m already… that’s terrible*.” (lines 364–365). The interaction then shifted — the doctor became “much nicer” once she understood the situation — but what the exchange makes transparent is the default assumption operative in the encounter: that a young woman at a fertility clinic could not possibly be there because of menopause. In Nora’s account, it is the patient who informs the doctor, who explains her own condition, and who does the work of making herself legible within a system that has no ready category for her. Nora’s observation — “*I noticed how many people have no understanding of why one might go to a fertility clinic, or that something like early menopause even exists*” (lines 366–367)— reads less as an individual complaint than as a diagnosis of systemic unpreparedness.

## Discussion

**Figure 1:**
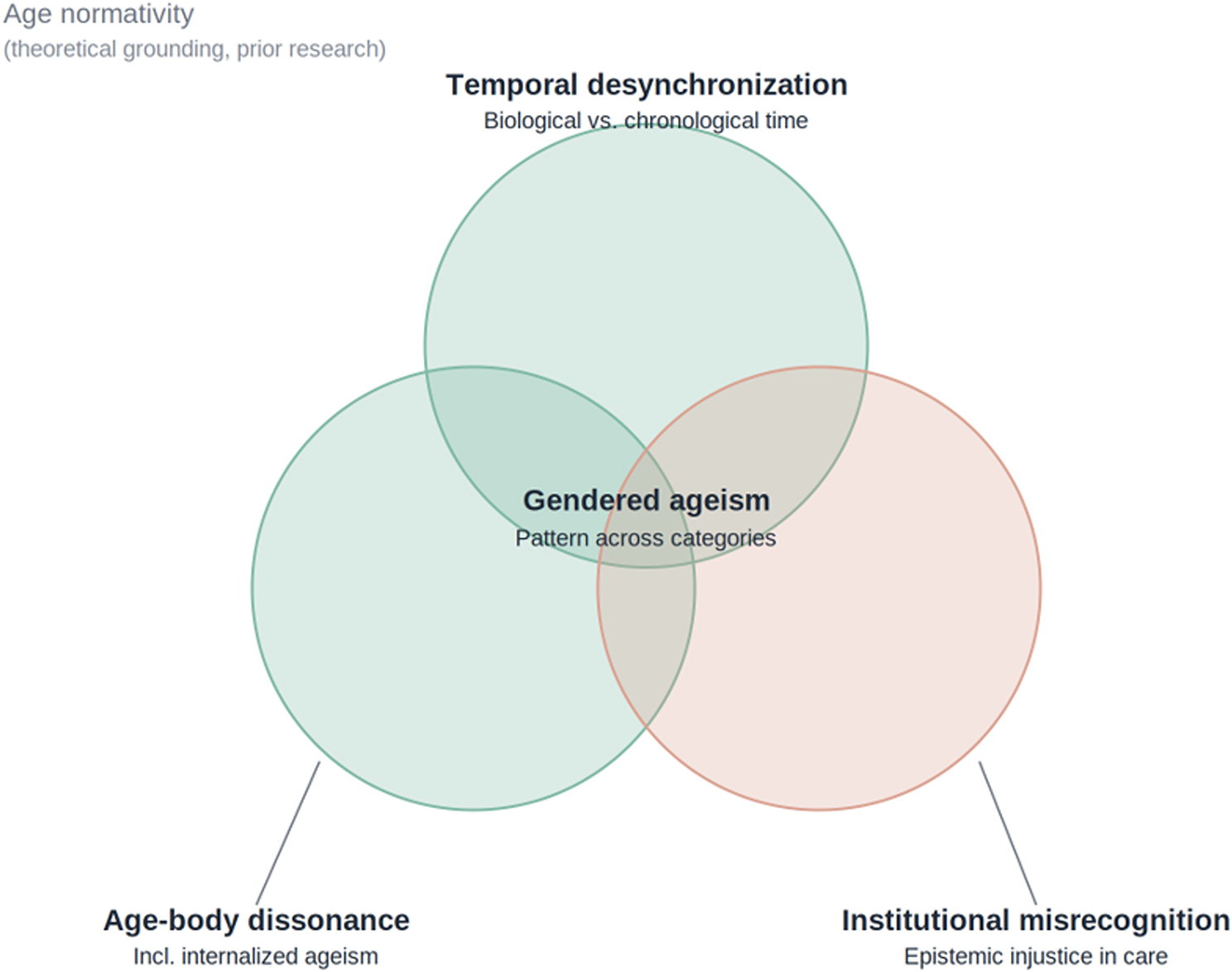
Fig. 1 Relationships among categories identified through the analysis

The following discussion situates the findings within the broader theoretical framework outlined above, examining how the experiences of women with POI reveal age-body dissonance and institutional misrecognition, and how these connect to the concept of gendered ageism. It moves from the social construction of menopause and its temporal dimensions, through the role of gender norms and bodily expectations, to the institutional mechanisms through which age- and gender-based misrecognition is reproduced within healthcare systems. The relationship between these categories is illustrated in Fig. 1. Gendered ageism is shown as the interpretative pattern emerging at the intersection of the three categories, not as an superordinate or causally prior category. Age normativity is shown as theoretical baclground, not as a fourth category.

### Navigating disrupted temporalities and age-body dissonance in premature ovarian insufficiency

This article has illuminated the profound impact of POI on women’s experiences of time, aging, and self-perception, demonstrating how the condition disrupts normative life trajectories. While the medical literature extensively details the diagnosis, management, and physical and emotional sequelae of POI [10, 2, 30], this research extends beyond these biomedical perspectives to explore the social and psychological dimensions of living with POI.

Beyond this general dissonance between bodily experience and chronological age, the data point to a more specific process of internalized ageism in Nora’s account. Her description of feeling ‘like a hundred-year-old, physically’ is telling not merely as an expression of distress, but in the cultural script it invokes: the hundred-year-old body she conjures is not one of accumulated experience or wisdom, but one collapsed entirely into pain, limitation, and non-function, precisely the deficit-oriented image of old age against which ageist discourse measures bodily worth. In applying this image to herself, Nora does not merely describe symptoms but reproduces a negative cultural evaluation of old age as a lens for interpreting her own body. This suggests that the age-body dissonance documented across participants can, under certain conditions, crystallize into a more specific internalization of ageist assumptions, rather than remaining a general unease about premature ageing.

### The social construction of menopause and the “sudden aging” phenomenon

What participants experience as an immediate ‘hormonal reality’ is not simply given, but produced through interpretive frameworks that link bodily changes to later life. The findings underscore that the passage of time plays a decisive role in shaping the experience of POI. For many women affected, a diagnosis of POI is accompanied by a feeling of having aged suddenly. This perception is heavily influenced by societal discourses that strongly associate menopause with the age range of 50 to 60 years [9]. When menopause occurs in a woman’s mid-20–30 s, this ingrained association imposes itself, creating a significant temporal incongruity. This finding resonates with earlier observations in the literature on premature menopause: “It is interesting to note that what the women described is the process of ageing rather than menopause per se. However, it does reflect the commonly held belief, particularly in the medical discourse, that menopause and ageing are synonymous. This belief perpetuates and strengthens the stereotypical image of menopausal women that impacts greatly on women’s experience.” (3:427).

### Challenging and expanding menopause discourses

The interview excerpts reveal that the intertwining of biomedical knowledge about menopause and their lived reality remains potent. Even when interviewees recognize that their experience as younger women with POI differs significantly from existing discourses on menopause, it proves difficult to fully detach from these dominant narratives. This suggests that a more expansive understanding of menopause is needed to accommodate diverse experiences. Previous research indicates that negative attitudes towards menopause are prevalent [32] and that societal myths significantly influence perceptions [33]. To foster greater diversity in the representation of menopause, feminist discourses addressing this life phase must be broadened. This includes giving greater consideration to the experiences of individuals who enter menopause early, trans* individuals, and intersectional perspectives that account for dimensions such as race and class. Expanding these discourses is therefore not only a cultural task but a healthcare imperative: without more inclusive and flexible understandings of menopause, healthcare systems risk systematically excluding those whose experiences fall outside dominant age frameworks.

### Temporal desynchronization and gendered life course expectations

Temporality and gendered life-course expectations intersect in this context. The study highlights the incongruity between chronological age and biological age, manifesting as temporal desynchronization. As Fuchs [31] argues, illness can disrupt not only the continuity of everyday life but the very experience of time itself, fragmenting the sense of a continuous self. This can be understood as a desynchronization of intersubjective time: participants experience their own temporal rhythm as falling out of step with that of their peers, whose youth remains legible and shared in a way that their own no longer is. This desynchronization is closely linked to normative, highly gendered expectations regarding life courses. The feeling of being “fast-forwarded” into a later life stage, while chronologically young, creates a unique form of vulnerability. As discussed above, this can—though not uniformly across participants—crystallize into internalized ageism, in which socially negative images of aging are applied to one’s own identity. This internal conflict arises from the dissonance between the inner self and the body’s state, challenging the expected sequence of life events and profoundly altering a person’s place in the life course. The emotional distress and social isolation experienced by women with POI are further exacerbated by these unmet expectations and the perceived loss of a “normal” trajectory [34].

Notably, this sense of premature aging was not confined to the youngest participants. Doreen, who was 40 at the time of the interview—at the upper boundary of the POI age range—also described bodily changes such as graying hair as occurring ‘too early.’ This suggests that the sense of prematurity is not simply a function of chronological age at diagnosis, but reflects a more fundamental shift in participants’ temporal frame of reference following the diagnosis itself, against which even ordinarily-timed bodily changes come to be read as out of time.

### Institutional misrecognition: structural dimensions of healthcare encounters

The findings of this study can be read through the concept of gendered ageism as a structural phenomenon—a form of bias embedded not only in individual attitudes but in the institutional arrangements, epistemic frameworks, and normative orders that shape healthcare and social life. Women with POI encounter a healthcare system that is structurally organized around normative age-gender assumptions: menopause is implicitly constructed as a concern of women in their fifties, rendering younger women with early menopause illegible within existing diagnostic and support structures. This invisibility appears not incidental but systemic, potentially reflecting the compounded operation of sexism and ageism within medical knowledge production, whereby women’s experiences are already subject to epistemic devaluation, and this devaluation intensifies when those experiences deviate from age-normative expectations.

Nora’s account of educating her own clinician illustrates this structural dimension particularly clearly. The astonishment it provokes is not reducible to age alone: it is precisely because young womanhood is culturally coded as a reproductive, fertility-oriented stage that early menopause becomes unintelligible within the clinical encounter. This is not attributable to the clinician’s medical competence in general, but to a frame of reference structured by normative expectations about whose body ages when. This inversion of epistemic roles—in which the patient carries the labor of making her condition legible—can thus be understood as a structural effect of gendered ageism: age-based misrecognition that is specifically shaped by normative assumptions about female reproductive temporality. This dynamic constitutes a form of epistemic injustice [35], in which patients are not recognized as credible knowers of their own condition and are instead required to compensate for gaps in clinical knowledge structured by these same gendered age norms.

What the narratives of women with POI suggest, then, is a particular mechanism of gendered ageism: the simultaneous imposition of age-associated stigma and the denial of age-appropriate support. As discussed above, this leaves them doubly marginalized—too young for existing menopause communities and support structures, yet unable to inhabit the social roles expected of their chronological age. Gendered ageism thus operates not only through overt discrimination, but through the very organization of what counts as a recognizable, legible, and treatable experience within healthcare systems—shaping access to diagnosis, the quality of care, and the recognition of patient experiences. Importantly, this suggests that addressing gendered ageism in healthcare requires more than increasing awareness among individual practitioners; it demands a critical examination of the normative assumptions embedded in medical knowledge, diagnostic criteria, and care infrastructures.

### Limitations

This study has several limitations. The sample comprised eight cisgender women recruited within a single national and cultural context, which limits the transferability of the findings to other settings and populations; in particular saturation cannot be claimed to the same extent for the more heterogeneous experiences within the sample, such as differences shaped by racialized identity, migration background, or socioeconomic position. Recruitment pathways may also have privileged women with greater familiarity with self-help structures or healthcare navigation, potentially underrepresenting women with more limited access to such resources. Coding and analysis were conducted by a single researcher; while reflexivity was supported through memo-writing and participation in analysis groups with colleagues, the absence of independent co-coding limits the extent to which analytic decisions can be externally verified.

Relatedly, as this analysis draws on a secondary re-examination of data originally collected for a broader doctoral study on temporality in POI, conceptual density was established for temporality as an overarching category in that original research; the specific pattern of gendered ageism identified within this material is accordingly best understood as an emergent, exploratory observation rather than one grounded in dedicated saturation.

Finally, as interviews were conducted in German and quotations translated into English for publication, some nuances of meaning, particularly around affectively and culturally specific expressions, may not be fully captured in translation. The cross-sectional design further means that the study captures participants’ age identities at a single point in time, rather than tracing how these may shift over the course of adjusting to the diagnosis. Relatedly, I note that gendered ageism is offered here as one productive interpretive lens among several that could plausibly illuminate these categories; the analysis does not foreclose complementary readings of the material.

## Conclusion

This study has examined how POI disrupts normative temporal frameworks through which women understand themselves, their bodies, and their place in the world. The findings show that this disruption is shaped by age norms, inflected by gender, operating both in individual self-perception and in the institutional structures of healthcare. Read through this lens, ageism here appears to manifest less as overt discrimination than as a pervasive process of misrecognition: of bodies that age ‘out of time,’ conditions that fall outside clinical expectation, and patients whose experiences do not fit the age-gender matrix organizing medical knowledge and care. These findings extend existing qualitative research on POI, which has predominantly emphasized fertility loss and psychological distress [7], by attending to age-related meaning-making. While grounded in a specific national and cultural context, the categories developed here may be relevant to other healthcare settings organized around comparable biomedical framings of menopause, though this would require further comparative research.

Clinically, these results call for greater structural attentiveness to women with POI: menopause guidelines, medical education, and healthcare policy should integrate age-divergent experiences like POI as integral rather than exceptional, with care moving beyond information provision toward validating patients’ experiences and supporting peer networks for younger women.

Future research should extend this qualitative, intersectional approach to trans* and non-binary individuals, for whom POI may carry additional and distinct dimensions of marginalization, and to the largely understudied dynamics of race, class, and disability in shaping access to diagnosis and care. More broadly, this study suggests that menopause should be understood not solely as a biomedical event, but as a socially situated experience shaped by age- and gender-based structural inequities.

## Data Availability

Data extracted from the articles included in the current study are available from the corresponding author upon reasonable request.

## Abbreviations

HRT: Hormone replacement therapy
POI: Premature ovarian insufficiency
